# Stepwise reduction of bone mineral density increases the risk of cage subsidence in oblique lumbar interbody fusion patients biomechanically: an in-silico study

**DOI:** 10.1186/s12891-022-06049-3

**Published:** 2022-12-12

**Authors:** Zhi-Qiang Yang, Ping Cai, Jing-Chi Li, Xian-Di Wang, Tian-Hang Xie, Xing-Xiao Pu, Run Lin, Jian-Cheng Zeng, Yue-Ming Song

**Affiliations:** 1 Department of Orthopedic Surgery and Orthopedic Research Institute, West China Hospital, Sichuan University, 37# Wuhou Guoxue road, Chengdu, 610041 Sichuan Province People’s Republic of China; 2 Department of Orthopedics, Affiliated Hospital of Nanjing University of Chinese Medicine, Nanjing, Jiangsu Province People’s Republic of China

**Keywords:** Oblique lumbar interbody fusion, Bone mineral density, Cage subsidence, Biomechanics, Finite element analysis

## Abstract

**Background:**

Cage subsidence causes poor prognoses in patients treated by oblique lumbar interbody fusion (OLIF). Deterioration of the biomechanical environment initially triggers cage subsidence, and patients with low bone mineral density (BMD) suffer a higher risk of cage subsidence. However, whether low BMD increases the risk of cage subsidence by deteriorating the local biomechanical environment has not been clearly identified.

**Methods:**

OLIF without additional fixation (stand-alone, S-A) and with different additional fixation devices (AFDs), including anterolateral single rod screws (ALSRs) and bilateral pedicle screws (BPSs) fixation, was simulated in the L4-L5 segment of a well-validated finite element model. The biomechanical effects of different BMDs were investigated by adjusting the material properties of bony structures. Biomechanical indicators related to cage subsidence were computed and recorded under different directional moments.

**Results:**

Overall, low BMD triggers stress concentration in surgical segment, the highest equivalent stress can be observed in osteoporosis models under most loading conditions. Compared with the flexion-extension loading condition, this variation tendency was more pronounced under bending and rotation loading conditions. In addition, AFDs obviously reduced the stress concentration on both bony endplates and the OLIF cage, and the maximum stress on ALSRs was evidently higher than that on BPSs under almost all loading conditions.

**Conclusions:**

Stepwise reduction of BMD increases the risk of a poor local biomechanical environment in OLIF patients, and regular anti-osteoporosis therapy should be considered an effective method to biomechanically optimize the prognosis of OLIF patients.

## Background

As a minimally invasive surgical method, oblique lumbar interbody fusion (OLIF) has been rapidly promoted, but corresponding studies that identify potential risk factors for complications are of great significance to optimize patients’ prognoses [1, 2]. The negative effect of cage subsidence on clinical outcomes is pronounced in OLIF patients. Specifically, the indirect decompression of nerve structures in OLIF patients is utterly dependent on interbody space distraction. Cage subsidence and the reduction of interbody space height could cause the recurrence of spinal canal stenosis and recompression of nerve structures [2, 3]. Risk factors for cage subsidence in OLIF patients have been widely reported; the deterioration of the local biomechanical environment initially triggers cage subsidence [4, 5].

Specifically, a longer OLIF cage (the OLIF cage crosses the epiphyseal ring) could reduce the risk of cage subsidence; corresponding biomechanical studies recorded a higher yield strength of the bony endplates (BEPs) in the longer OLIF cage group [6, 7]. Moreover, although the safety and effect of stand-alone (S-A) OLIF operations (i.e., without any additional fixation devices (AFDs)) have been validated, AFDs are still widely used in OLIF operations to optimize instant postoperative stability and reduce the risk of cage migration and surgical segmental instability [2, 6]. Studies have also proven that different AFDs might also affect the local biomechanical environment on the fusion segment [6, 8, 9]; corresponding clinical follow-up recorded different cage subsidence incidence rates in OLIF patients fixed by different AFDs. In summary, the mechanism for this complication should be well explained biomechanically, and the identification of postoperative biomechanical changes is of great significance for better understanding the pathological mechanism of cage subsidence.

The reduction in bone mineral density (BMD) has been repeatedly proven to be an initial trigger for cage subsidence [10, 11]. Significantly, during the pathological process of osteoporosis, the yield strength of bony structures dramatically decreases [12, 13]. Thus, the risk of failure of BEPs will increase under the same grade of stress concentration. Additionally, it is worth noting that the decrease in the elastic modulus for bony structures may also affect the load transmission pattern after lumbar operations [14, 15]. In other words, this change may lead to stress concentration on the surgical segment and increase the risk of cage subsidence.

On the basis of the above theoretical and clinical foundations, we hypothesized that the relationship between poor BMD and a higher risk of cage subsidence may not be limited to a decrease in bony yield strength but also rooted in changes in the local biomechanical environment, and this relationship may be affected by different AFDs. However, to the best of our knowledge, no published studies have elucidated this topic. In this study, to illustrate the biomechanical effects of BMD reduction in OLIF patients with different AFDs, we performed surgical simulations in an anterior constructed and validated lumbosacral (L3 to S1) finite element (FE) model. Stress distribution in cranial and caudal sides BEPs, the OLIF cage, and AFDs was computed and recorded.

## Methods

### Construction and validation of the intact model

Our previously published studies have presented the construction strategy for the intact FE model used for this study [16–18]. Bony structures from L3 to S1 have been constructed in this model. We constructed bony structures in 3D-CAD software by drawing smoothed surfaces. This method can replace irregular structures from the bony model directly reconstructed based on imaging data. In the construction of bony structures, the cortical shell and cancellous core were constructed separately, and the construction of BEPs was independent of other cortical bone. Based on the measurement of CT imaging data, the concave angles and depth of different segments’ BEPs in this FE model have been set to be the equal value of imaging data measurement [16–18].

This study’s construction of nonbony structures was also consistent with our published studies [16–18]. During intervertebral disc (IVD) construction, the surrounding annulus and central nucleus were separately constructed. The cross-sectional area’s ratio nucleus was defined based on the measurement of MRI imaging data. In this process, the cross-sectional area of the nucleus was set as 38% of the IVD, and the average radius of the IVD was set as 95.5% of the vertebral body’s average radius [16–18]. Fact cartilages of zygapophyseal joints (ZJs) were set as contact surfaces, and the contact type between cartilages was defined as frictionless [19, 20]. Moreover, seven different ligamentum structures, including the anterior longitudinal ligament (ALL), posterior longitudinal ligament (PLL), ligamentum flavum (LF), inter transverse process ligament (ITL), super spinous process ligament (SSL), inter spinous process ligament (ISL), and capsule of ZJs, were constructed separately with different cross-sectional areas in the preprocessing process of the FE [19, 20].

To verify whether the current FE model could make a real representation of the biomechanical environment, multi-indicator model validation was performed by comparing the differences between the computed indicator values with the in vitro measured average values in our previously published studies. In this process, the range of motions (ROMs), intradiscal pressure (IDP), disc compression (DC) and contact force of the ZJs have been computed [16–18]. The computational results show that the differences between the computed and measured values were less than one standard deviation; this result indicates that the current FE model could make good representation of the acute lumbar biomechanical environment, and surgical simulations can be performed based on this model.

### Simulations of OLIF with different AFDs in models with different elastic moduli

**Fig. 1:**
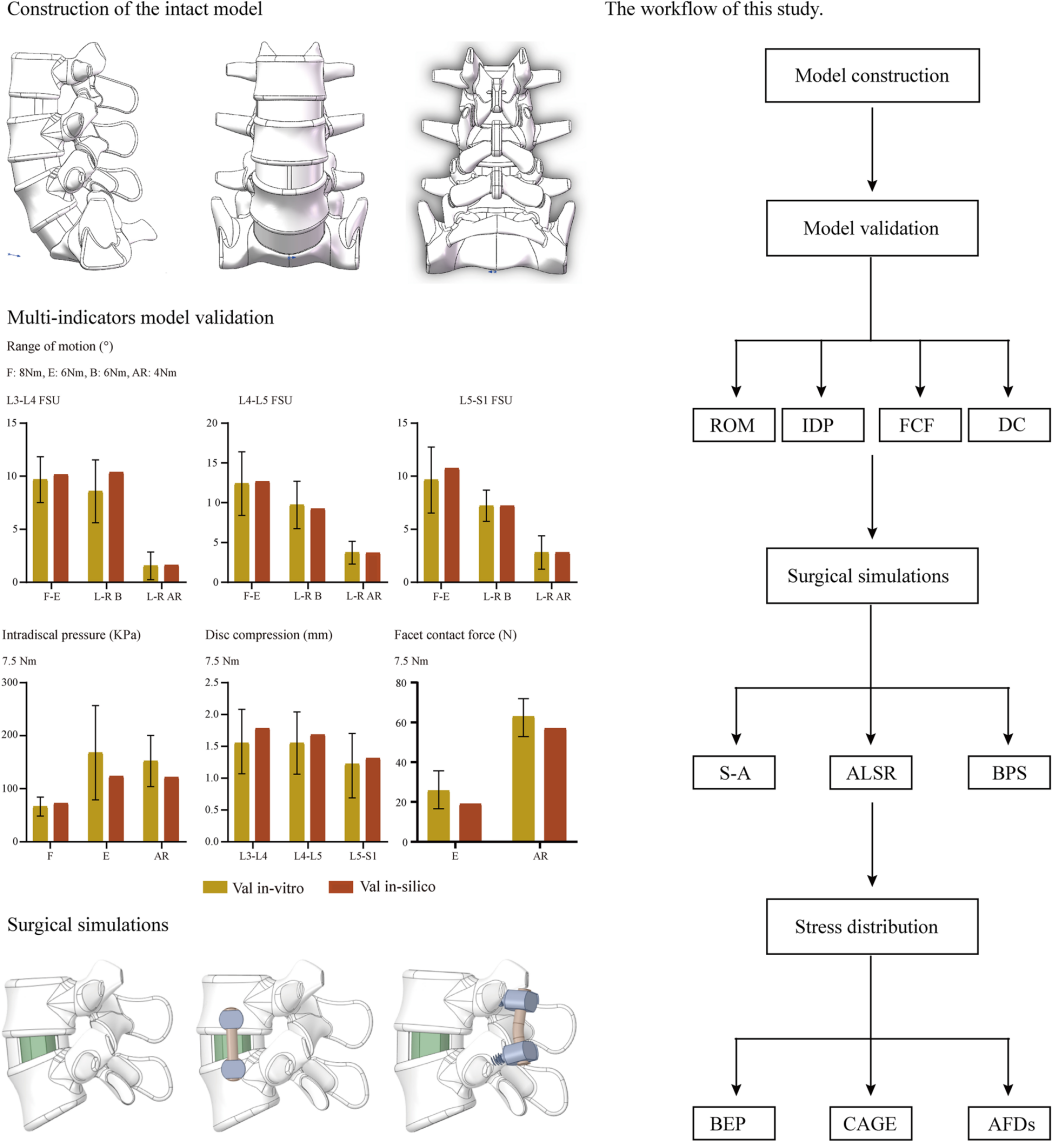
Schematics for the workflow (model construction, validation, and surgical simulations) in this study; Abbreviations: FSU = function spinal unit; F-E = flexion and extension; L-R B = left and right bending; L-R AR = left and right axial rotation; ROM = range of motion; IDP = intradiscal pressure; FCF = facet contact force; DC: disc compression; S-A = stand-alone; ALSR = anterolateral single rod; BPS = bilateral pedicle screw; BEP = bony endplate; AFDs = additional fixation devices

The L4-L5 motion segment was selected to simulate OLIF with different AFDs. This model construction strategy was rooted in the high incidence rate of disc degeneration in this motion segment [16, 17]. As a result, OLIF is most prevalent in the L4-L5 segment. A full-size OLIF cage model 50 mm in length and 18 mm in width was constructed in the same 3D-CAD software. After cage insertion, the postoperative interbody space was simulated as follows: First, to simulate the discectomy, two lateral parts of the annulus were deleted, and all of the nucleus was also removed (Fig. 1). During the simulation of endplate preparation, the cranial and caudal side cartilage endplates were removed, and the BEPs were preserved [16, 17]. The lordotic angle and disc height of the OLIF models were identical to the preoperative model to eliminate the mechanical effects of sagittal alignments and disc distraction changes [16, 17].

Moreover, the simulation of AFDs occurred as follows: In the anterolateral single rod screws (ALSRs) fixation simulation, two screws were inserted into the vertebral bodies of the L4-L5 motion segment. The deep screws were set to penetrate the contralateral cortex. The screw axes in the transverse plane were parallel to the long axis of the OLIF cage, which was parallel to the corresponding BEPs in the coronal plane. When simulating bilateral pedicle screws (BPSs) fixation, four cannulated pedicle screws whose diameter was the same as the ALSRs were inserted into the vertebral bodies. The screw axes in the transverse plane were parallel to the pedicle, which was identical to that of the ALSRs fixation in the coronal plane [16, 17, 21]. The connection between the screw tulip and the nut was simplified to increase the computational efficiency in the ALSRs and BPSs simulations; screw compaction effects were simulated by adjusting the material property of the compaction area according to corresponding studies (Fig. 1) [16, 17, 21]. Finally, in regard to the construction of models with different BMDs, the material properties of bony structures, including the cortical, cancellous, and BEP structures, were adjusted. The elastic modulus of these bony structure steps was reduced to simulate patients with osteopenia and osteoporosis in the preprocessing of the FE models. In this process, consistent with published studies, the morphological parameters of the models were kept identical [17, 22].

### Boundary and loading conditions

This study’s boundary and loading conditions were identical to those in our published studies [16, 17, 21]. We completely restricted the inferior side of S1’s freedom degree and applied different directional moments, including 8 Nm flexion, 6 Nm extension and bending, and 4 Nm axial rotation on the superior side of L3 [23–25]. In this process, S-A and BPSs models were symmetrical to the sagittal plane; thus, bending and axial rotation loading conditions were computed merely on the left side [21, 26]. In contrast, the ALSRs model was not symmetrical along the sagittal plane, so bending and rotation loading conditions were computed under both the left and right sides.

To eliminate the confounding effect of mesh sizes on the computational results, a mesh convergence test was performed in the preoperative intact model. In this process, the IDP of the L4-L5 segment was repeatedly computed and recorded in the model with different mesh sizes. The model was considered converged if the change in the computed IDP was less than 3%. The mesh sizes of different components are presented in Table 1. Moreover, given that cage subsidence commonly occurs in the instant postoperative period, corresponding contact types were defined according to published studies. In this process, the frictional coefficients between cage-BEPs, grafted bone-BEPs and bone-screw interfaces were set as 0.2, 0.46, and 0.2, respectively [16, 17, 21]. The material properties of the components are presented in Table 1.

**Table 1:** Material properties of FE models’ components

Components	Elastic modulus (MPa)	Poisson’s ratio	Cross-section (mm^2^)	Mesh sizes (mm)	References
Bony structures					
Cortical (Normal BMD)	E_xx_ = 11,300 E_yy_ = 11,300 E_zz_ = 22,000 G_xy_ = 3800 G_yz_ = 5400 G_xz_ = 5400	V_xy_ = 0.484 V_yz_ = 0.203 V_xz_ = 0.203	/	1.7	[14, 17]
Cancellous (Normal BMD)	E_xx_ = 140 E_yy_ = 140 E_zz_ = 200 G_xy_ = 48.3 G_yz_ = 48.3 G_xz_ = 48.3	V_xy_ = 0.45 V_yz_ = 0.315 V_xz_ = 0.315	/	2.6	[14, 17]
Bony endplates (Normal BMD)	12,000	0.3	/	1.2	[14, 17]
Cortical (Slight reduction of BMD)	Exx = 9436 Eyy = 9436 Ezz = 18,370 Gxy = 3173 Gyz = 4509 Gxz = 4509	Vxy = 0.484 Vyz = 0.203 Vxz = 0.203	/	1.7	[14, 17]
Cancellous (Slight reduction of BMD)	Exx = 93.8 Eyy = 93.8 Ezz = 150 Gxy = 32.36 Gyz = 36.23 Gxz = 36.23/	Vxy = 0.45 Vyz = 0.315 Vxz = 0.315	/	2.6	[14, 17]
Bony endplates (Slight reduction of BMD)	10,035	0.3	/	1.2	[14, 17]
Cortical (Significant reduction of BMD)	Exx = 7571 Eyy = 7571 Ezz = 14,740 Gxy = 2546 Gyz = 3618 Gxz = 3618	Vxy = 0.484 Vyz = 0.203 Vxz = 0.203		1.7	[14, 17]
Cancellous (Significant reduction of BMD)	Exx = 47.6 Eyy = 47.6 Ezz = 100 Gxy = 16.42 Gyz = 24.15 Gxz = 24.15	Vxy = 0.45 Vyz = 0.315 Vxz = 0.315	/	2.6	[14, 17]
Bony endplates (Significant reduction of BMD)	8070	0.3	/	1.2	[14, 17]
Non bony structures					
Annulus	Hypoelastic material		/	2.0	[18, 26]
Nucleus	1	0.49	/	1.7	[18, 26]
Cartilages	10	0.4		0.7	[18, 26]
Ligaments					
Anterior longitudinal ligaments	Calibrated load-deformation curved under different loading conditions	0.3	60	/	[16, 17]
Posterior longitudinal ligaments	Calibrated load-deformation curved under different loading conditions	0.3	21	/	[16, 17]
Ligamentum flavum	Calibrated load-deformation curved under different loading conditions	0.3	60	/	[16, 17]
Interspinous ligaments	Calibrated load-deformation curved under different loading conditions	0.3	40	/	[16, 17]
Supraspinous ligaments	Calibrated load-deformation curved under different loading conditions	0.3	30	/	[16, 17]
Intertransverse ligaments	Calibrated load-deformation curved under different loading conditions	0.3	10	/	[16, 17]
Capsular	7.5 (≤25%) 32.9 (>25%)	0.3	67.5	/	[16, 17]
Internal fixation devices					
PEEK OLIF Cage	3500	0.3	/	2.0	[16, 17]
Titanium alloy screw	110,000	0.3	/	0.5	[16, 17]

*Abbreviations*: *FE* Finite element, *BMD* Bone mineral density, *PEEK* Polyetheretherketone, *OLIF* Oblique lumbar interbody fusion

## Results

### Indicator selection and stress distribution in BEPs

**Fig. 2:**
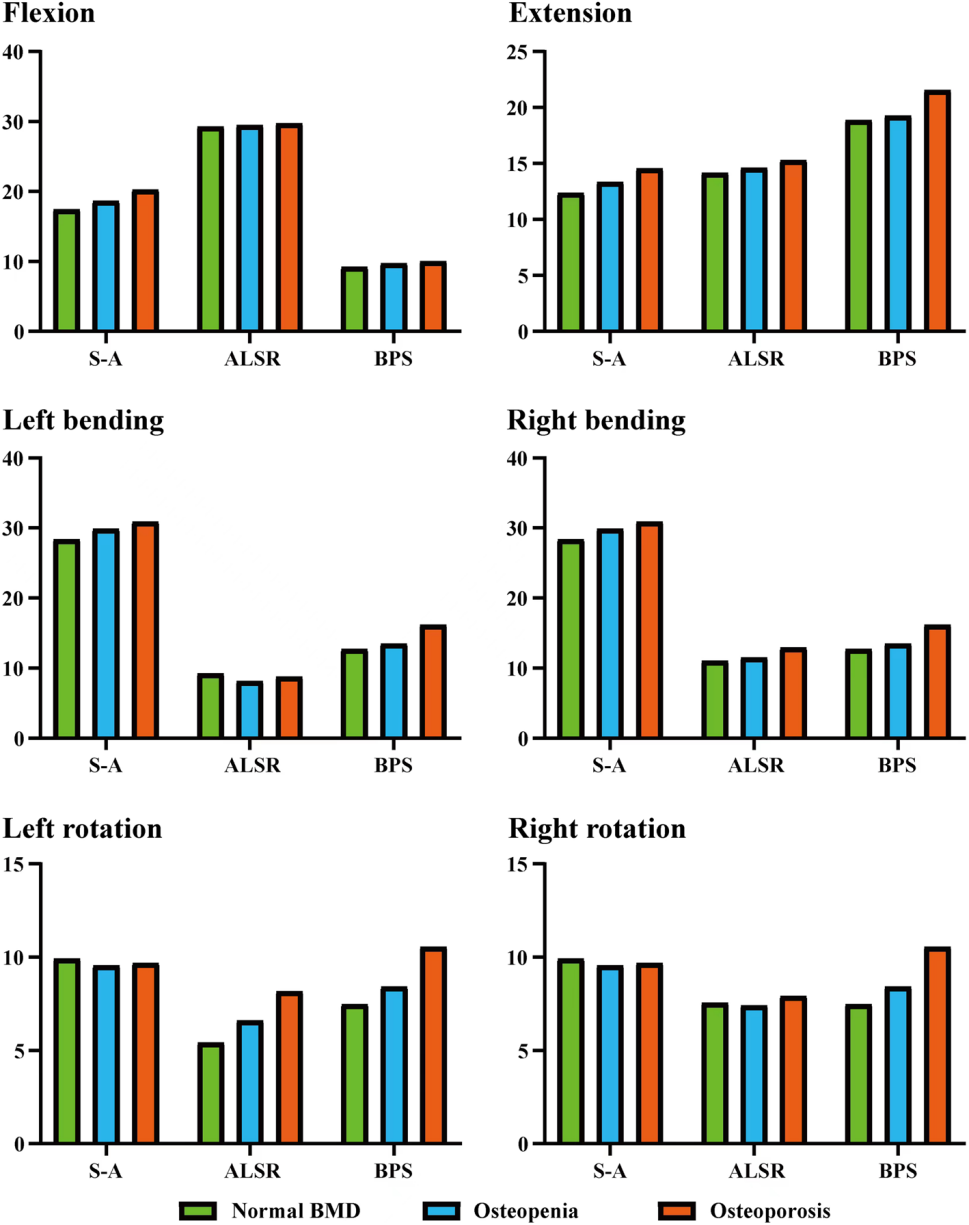
Variations in the superior bony endplate (BEP) maximum stress (MPa); Abbreviations: S-A = stand-alone; ALSR = anterolateral single rod; BPS = bilateral pedicle screw; BMD = bone mineral density

**Fig. 3:**
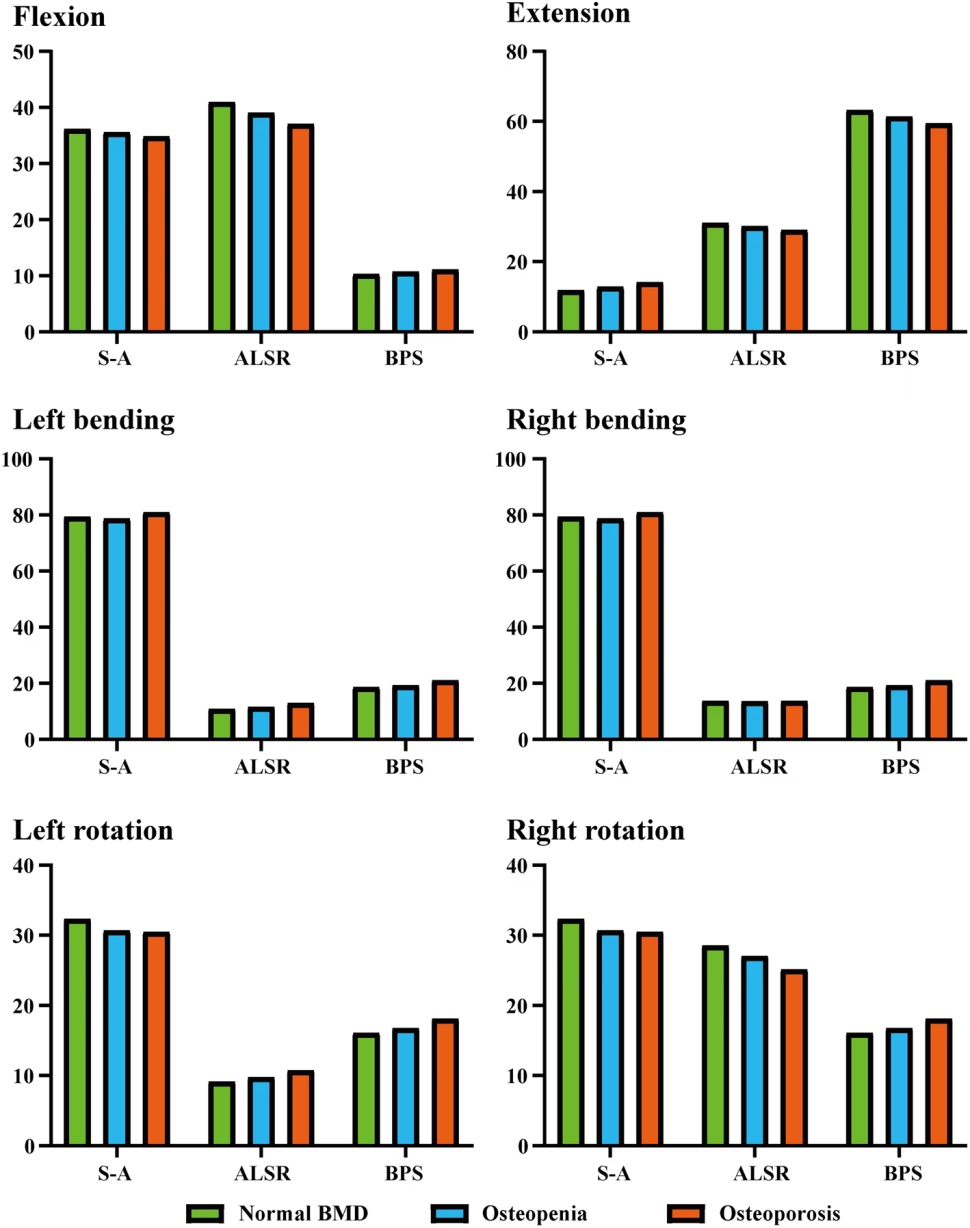
Variations in the inferior bony endplate (BEP) maximum stress (MPa); Abbreviations: S-A = stand-alone; ALSR = anterolateral single rod; BPS = bilateral pedicle screw; BMD = bone mineral density

**Fig. 4:**
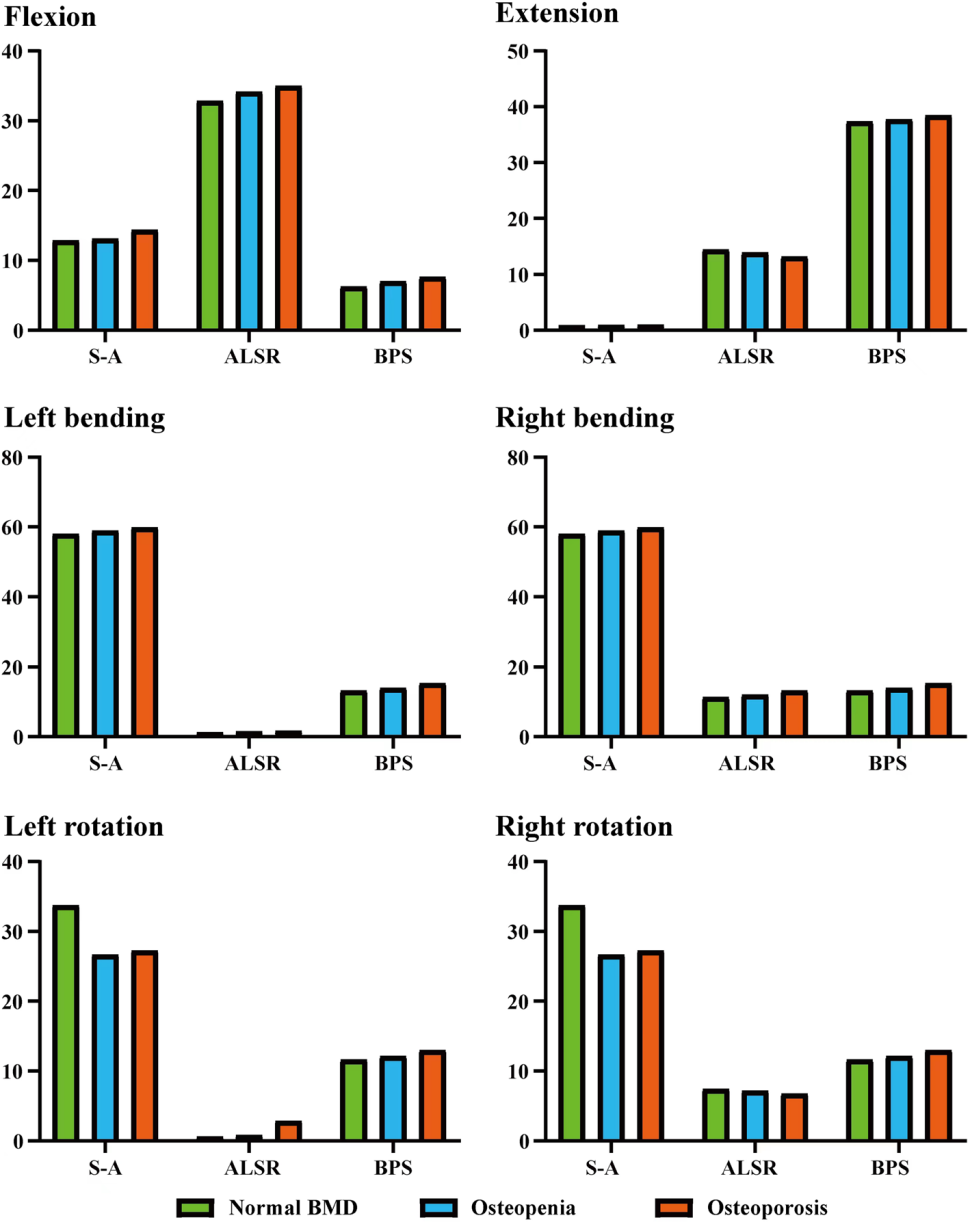
Variations in the oblique lumbar interbody fusion (OLIF) cage maximum stress (MPa); Abbreviations: S-A = stand-alone; ALSR = anterolateral single rod; BPS = bilateral pedicle screw; BMD = bone mineral density

**Fig. 5:**
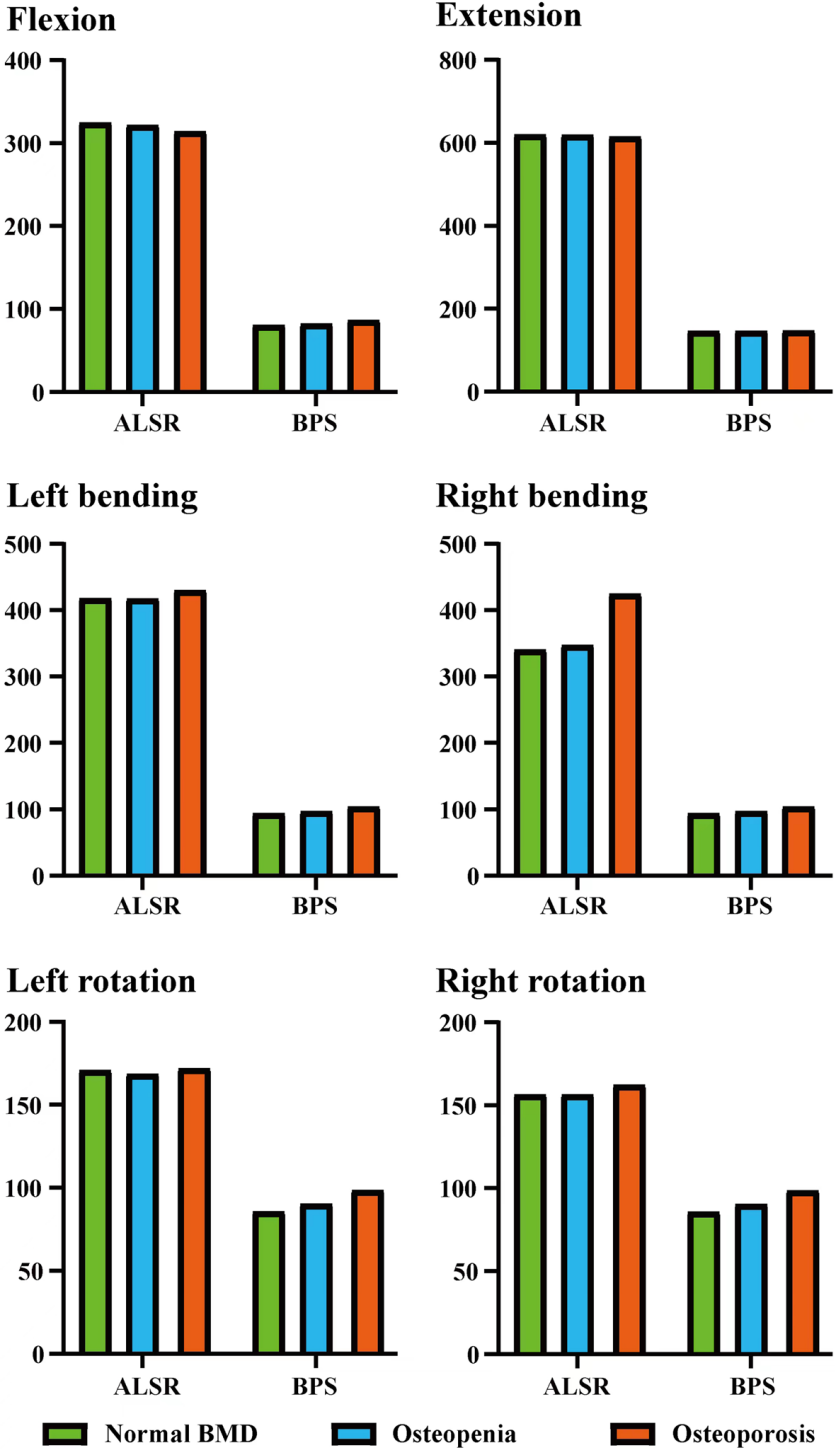
Variations in the different additional fixation devices (AFDs) maximum stress (MPa); Abbreviations: ALSR = anterolateral single rod; BPS = bilateral pedicle screw; BMD = bone mineral density

Given that BEP fracture is the main pathological process for cage subsidence and that stress distribution patterns mainly affect the risk of BEP fracture, this study computed and recorded the maximum stress of cranial and caudal side BEPs, OLIF cages, and AFDs (Figs. 2, 3, 4 and 5). The computational results showed that a clear variation tendency was observed in the models with different BMDs. Specifically, the maximum value of the superior BEP von Mises stress step increased with the decrease in the bony elastic modulus under almost all of the loading conditions (Fig. 2). The maximum variation tendency was observed in the ALSRs and BPSs models; compared with the postoperative model with normal BMD, the maximum stress values increased 12.45 and 41.1% in the osteopenia and osteoporosis models under the axial rotation loading condition of the models fixed by BPSs and that of ALSRs models increased 22.22 and 50.23%, respectively. In contrast, the opposite variation tendency was observed in the S-A models under the same loading conditions. The maximum stress values of BEPs decreased 3.64 and 2.22% in models with step reduction of BMD.

**Fig. 6:**
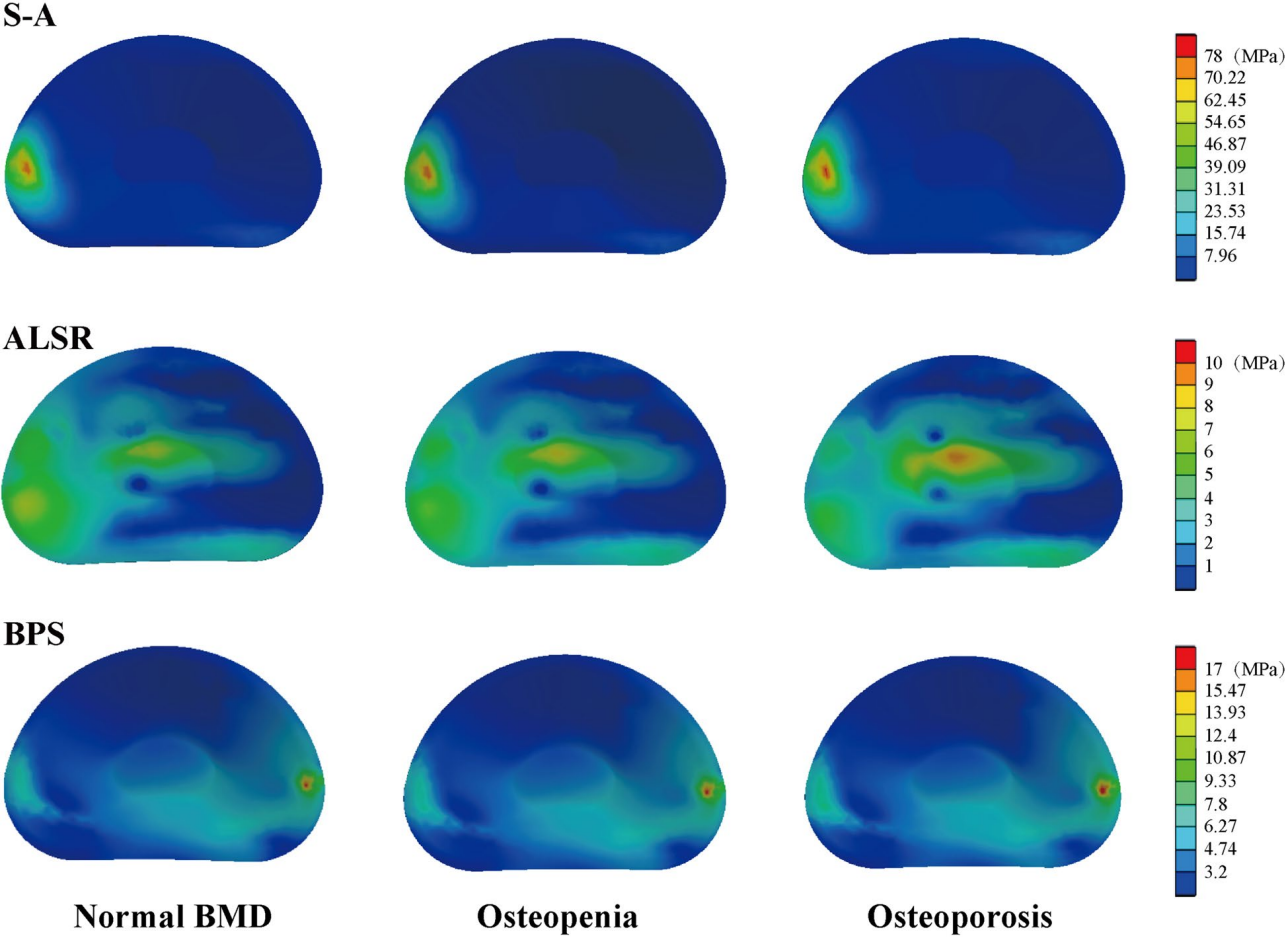
Nephograms for the inferior bony endplate (BEP) under left bending loading; Abbreviations: S-A = stand-alone; ALSR = anterolateral single rod; BPS = bilateral pedicle screw; BMD = bone mineral density

Although the overall variation trend of the stress distribution in the inferior BEP is the same as that in the superior BEP, some differences still existed (Figs. 3 and 6). Specifically, in the S-A model with osteopenia, the maximum stress of the inferior BEP increased only under the extension loading condition compared with the model with normal BMD. In the model with a further decrease in BMD (i.e., osteoporosis model), the maximum stress increased under extension and bending loading conditions and decreased under flexion and axial rotation loading conditions. The most significant increase was close to 20% in the extension model, while the largest decline was just over 5% with axial rotation. Similarly, in the ALSRs model with osteopenia, the inferior BEP’s maximum stress increased 5.63% under the left lateral bending loading condition, which decreased in different grades under the other five loading conditions. In the osteoporosis model fixed by ALSRs, the maximum stress increased by approximately 18% in both the left bending and axial rotation loading conditions, but decreased in the other four loading conditions. Under the right axial rotation loading condition, the largest decrease was nearly 12%.

AFDs also affected local load transmission patterns and the resulting risk of cage subsidence. Under bending and axial rotation loading conditions, ALSRs and BPSs decreased the maximum stress of both cranial and caudal BEPs, and the decrease degree of ALSRs was larger than that of BPSs. In contrast, under the extension loading condition, the maximum stress increased with AFDs, which was higher in the BPSs models than in the ALSRs models. The biomechanical changes in flexion loading conditions were different from that in the other loading conditions. ALSRs fixation increased the maximum stress value, but the value decreased in the BPSs fixed models.

### Stress distribution in the OLIF cage

**Fig. 7:**
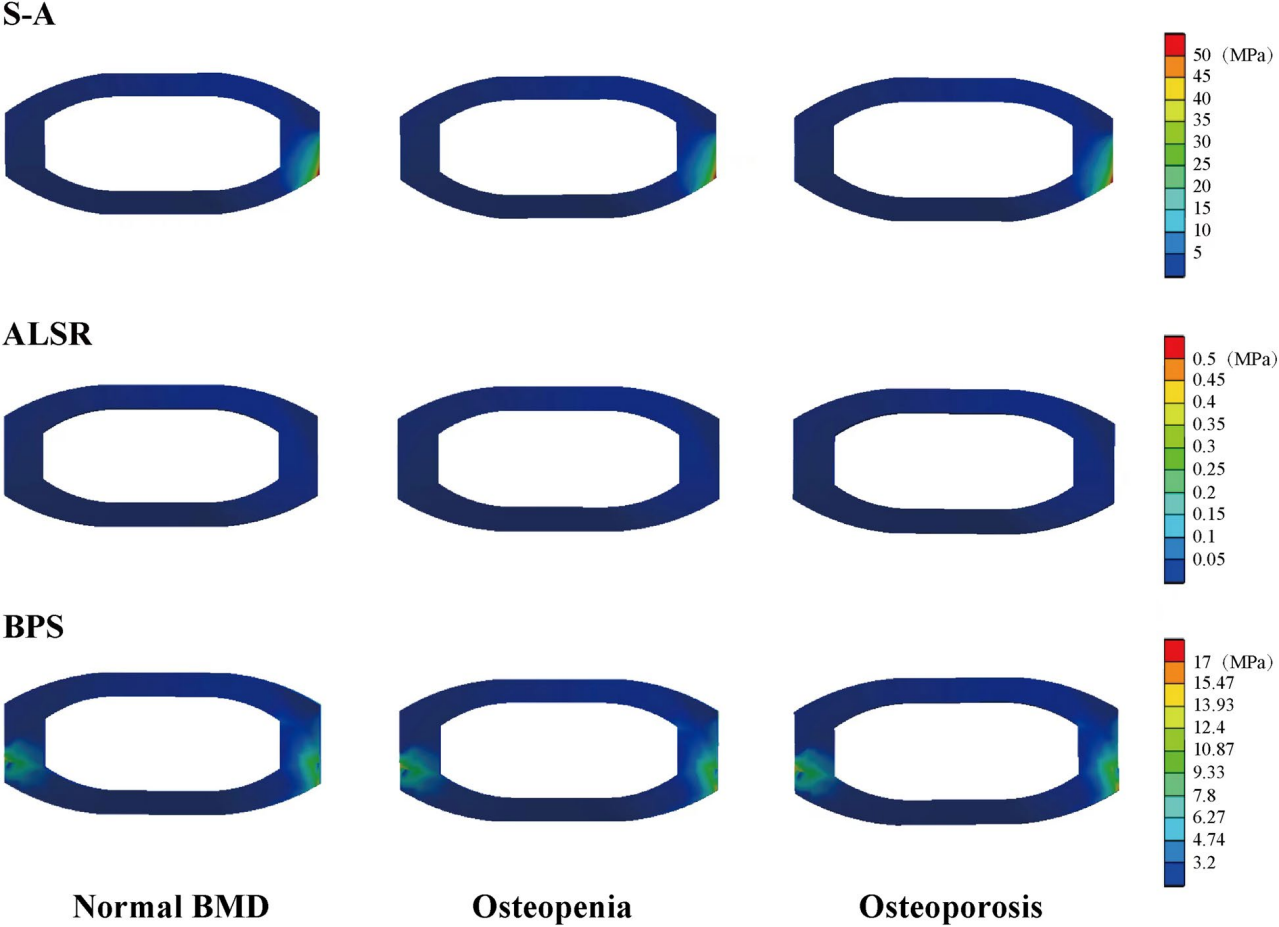
Nephograms for the oblique lumbar interbody fusion (OLIF) cage under left bending loading; Abbreviations: S-A = stand-alone; ALSR = anterolateral single rod; BPS = bilateral pedicle screw; BMD = bone mineral density

The overall variation tendency of the OLIF cage’s maximum stress was consistent with BEPs (i.e., the maximum stress step increased with the decrease in BMD). Specifically, the maximum stress of the OLIF cage increased under most of the loading conditions. In the S-A models, compared with the model with normal BMD, the maximum stress of the OLIF cage increased by nearly 40% under the extension loading condition, but decreased by approximately 20% under the axial rotation loading condition. In the models fixed by ALSRs, an approximately 4 and 10% reduction of maximum stress was observed in the extension and axial rotation loading conditions in the osteopenia and osteoporosis models, respectively. In contrast, in the models fixed by BPSs, the cage’s maximum stress step increased under all of the loading conditions. When comparing the difference in stress distribution in the models with different AFDs, the highest stress values in the S-A models were observed under the bending and axial rotation loading conditions. The stress value was highest in the BPSs models under extension and in the ALSRs model under the flexion loading condition. Moreover, it is worth noting that the OLIF cage’s stress values under the extension loading condition in the S-A models and under left side bending and axial rotation loading conditions were lower than those of the others (lower than 1 MPa) (Figs. 4 and 7).

### Stress distribution in AFDs

We also compared the stress distribution in different AFDs (i.e., in ALSR and BPS systems) in models with different BMDs. The highest stress in AFDs was observed under the extension loading condition, and that in the ALSRs models was higher than that in the BPSs models under all loading conditions. The variation tendencies in the ALSRs and BPSs models were different. Specifically, the overall tendency of stress distribution changes in the BPSs models was identical to that in the BEPs and OLIF cages. The maximum stress of the BPSs steps increased with decreases in BMD under all loading conditions. In contrast, in the ALSRs models, as BMD decreased, the maximum stress steps increased only under the right bending and rotation loading conditions; in the osteoporosis model, the stress value increased by nearly one-fourth under the right bending loading condition (Fig. 5).

## Discussion

In this study, to verify if stepwise reduction of BMD increases the risk of cage subsidence in OLIF patients biomechanically, OLIF models with different AFDs and bony elastic modulus have been constructed. Stress values related to cage subsidence have been computed and recorded. Computational results recorded higher stress values with step reduction of bony elastic modulus. Therefore, we can conclude that patients with poor BMD suffer a higher risk of cage subsidence for poor postoperative local biomechanical environments.

By measuring the BMD of vertebral bodies in the fusion segment and testing the maximum load of BEP-cage interfaces, studies have proven that osteoporosis is a significant risk factor for cage subsidence by reducing the yield strength of bony structures [7, 27]. However, studies have reported that stress distribution pattern changes in the fusion segment also affect the risk of cage subsidence. The biomechanical significance of several factors, including the screw position and different types of AFDs, on BPEs and interbody cages have been clearly investigated [6, 28, 29]. The deterioration of the stress distribution pattern on BEPs and cages biomechanically increases the risk of BEP fracture and the resulting cage subsidence. The computational results of these studies could provide theoretical guidance for better understanding the pathological process of cage subsidence and optimizing patient treatment strategies.

As mentioned above, the negative clinical effects of stepwise BMD reduction on the risk of cage subsidence have been widely reported. However, based on the current computational results, we believe that the relationship between poor BMD and a higher risk of cage subsidence is not limited to the decrease in the strength of the bony structures but also rooted in postoperative stress concentration in the surgical segment structures caused by a reduction in the bony elastic modulus. To verify this hypothesis, OLIF models with different BMDs (i.e., different elastic moduli) were constructed, and the stress distribution patterns in the fusion segment were computed and recorded. The computational results showed that poor BMD will lead to stress concentration in the surgical segment and that the use of AFDs does not dramatically change this variation tendency under most loading conditions. To the best of our knowledge, this is the first study to identify the interaction between different BMDs and AFDs. Based on the current computational results, this study proves that regardless of the kind of AFDs used, BMD reduction increases the risk of cage subsidence biomechanically. Therefore, we believe that regular anti-osteoporosis treatment should be promoted in OLIF patients with osteoporosis. The clinical significance of this treatment strategy is not only to increase the bone quality but also to optimize the local biomechanical environment.

FE models, rather than fresh specimen biomechanical tests, were selected in this study. This experimental strategy’s reasons are presented as follows: First, as a sensitivity analysis, biomechanical changes in models with different BMDs fixed by different AFDs were computed in this study. Meanwhile, studies have shown that morphology parameters, alignments, and even the insertional angles of AFDs affect the local stress distribution. If we want to control the confounding effects of these factors, a large sample size mechanical test is necessary. However, fresh specimens are scarce, and it is difficult to obtain enough fresh specimen samples to perform the same experimental strategy. In contrast, a well-validated FE model can be repeatedly used without any additional expense, and the biomechanical effects of confounding effects can be eliminated entirely [27, 30–32]. Moreover, in in vitro biomechanical tests, the mechanical sensor cannot be inserted into the space between the OLIF cage and BEPs. As a result, the stress distribution cannot be directly measured. Nevertheless, the FE model can directly compute the stress distribution in both structures in the interbody space and AFDs [27, 31, 33]. Considering that the FE model with validation can represent the natural biomechanical environment, we believe that the computational results from the current FE models are reliable and could provide theoretical guidance for clinical practice.

Traditionally, BPSs is widely seen as the gold standard for AFDs in LIF patients. In OLIF surgery, percutaneous insertion of BPSs is also widely used. However, this surgical procedure requires an intraoperative change in body position, prolongs the operative time, and increases the patient’s blood loss [4, 28, 34, 35]. In contrast, ALSRs fixation can be performed in the same surgical incision without any body position changes. This surgical method is convenient without body position changes and repeats intraoperative radiolucency in the percutaneous BPSs incision. Therefore, ALSRs fixation is an alternative method to replace BPSs fixation in OLIF patients. However, based on the current study’s computational results, the maximum stress of ALSRs fixation was higher than that of BPSs fixation under all loading conditions. Stress concentration in AFDs increases the risk of fixation failure, micro-damage of the screw compaction area, and the resulting screw loosening. Screw loosening is subsequently related to patients’ poor prognoses. Therefore, we believe further optimization of ALSRs fixation and related surgical methods is necessary for our future work.

Indeed, this study still has inherent limitations. First, morphological changes during the reduction of BMD were eliminated in this study. This is because there is no commonly used model construction strategy, and we could perform accurate model calibration and validation if we casually adjusted the morphological parameters [14, 22]. In addition, regional differences in cancellous bone have also been proven by published mechanical tests and imaging measurements [36, 37]. However, regional differences in the cancellous areas were also ignored in this study for the same reason. To further increase the computational accuracy, a large sample imaging measurement should be performed, and model construction should be optimized based on these measured values in our future work. Moreover, as a preliminary study, this study has been accomplished in a single model without clinical evidence and statistical significance, and only stress values in a single model can be recorded in this study. Therefore, current computational results and corresponding conclusions should be reverified in our future studies.

## Conclusions

A decrease in BMD triggers stress concentration in the surgical segment. Thus, the reasons for the higher risk of cage subsidence in patients with poor BMD are not limited to the poor yield strength of bony structures. In addition, the potential risk of screw loosening for ALSRs is higher than that for BPSs in OLIF patients, and the ALSRs system should be further modified to optimize the postoperative biomechanical environment.

## Data Availability

All the data of the manuscript are presented in the paper.

## Abbreviations

OLIF: Oblique lumbar interbody fusion
BMD: Bone mineral density
S-A: Stand-alone
AFDs: Additional fixation devices
ALSRs: Anterolateral single rod screws
BPSs: Bilateral pedicle screws
BEPs: Bony endplates
LIF: Lumbar interbody fusion
FE: Finite element
IVD: Intervertebral disc
ZJs: Zygapophyseal joints
ALL: Anterior longitudinal ligament
PLL: Posterior longitudinal ligament
LF: Ligamentum flavum
ITL: Inter transverse process ligament
SSL: Super spinous process ligament
ISL: Inter spinous process ligament
ROMs: Range of motions
IDP: Intradiscal pressure
DC: Disc compression
FSU: Function spinal unit
F-E: Flexion and extension
L-R B: Left and right bending
L-R AR: Left and right axial rotation
FCF: Facet contact force
